# Supplementary material for: Prevalence of malocclusion and occlusal traits in the early mixed dentition in Shanghai, China
Source: PeerJ. 2019 Apr 2;7:e6630. doi: 10.7717/peerj.6630 (PMC6450371; doi:10.7717/peerj.6630)
Supplement: Table S1 [file peerj-07-6630-s002.docx]

**Table S1**. Prevalence of severe malocclusion in in 7-9-years-old children in Shanghai

|  | n | Non-severe malocclusion | |  | Severe malocclusion ^a^ | | *P* |
| --- | --- | --- | --- | --- | --- | --- | --- |
|  |  | n | % |  | n | % |  |
| Age (years) |  |  |  |  |  |  | 0.080 ^b^ |
| 7 | 937 | 715 | 76.3 |  | 222 | 23.7 |  |
| 8 | 1217 | 888 | 73.0 |  | 329 | 27.0 |  |
| 9 | 656 | 470 | 71.6 |  | 186 | 28.4 |  |
| Gender |  |  |  |  |  |  | 0.349 ^b^ |
| Boys | 1479 | 1102 | 74.5 |  | 377 | 25.5 |  |
| Girls | 1331 | 971 | 73.0 |  | 360 | 27.0 |  |
| Total | 2810 | 2073 | 73.8 |  | 737 | 26.2 |  |

a: Who exhibited one or more of the following conditions were registered as severe malocclusion: Angle’s Class III, increased overjet > 8 mm, anterior crossbite, open bite > 3 mm, posterior crossbite, scissors bite, and anterior or posterior crowding > 4 mm.

b: chi-squared test.
